# Exploring the Determinants of Color Perception Using #Thedress and Its Variants: The Role of Spatio-Chromatic Context, Chromatic Illumination, and Material–Light Interaction

**DOI:** 10.1177/0301006620963808

**Published:** 2020-11-12

**Authors:** Stacey Aston, Kristina Denisova, Anya Hurlbert, Maria Olkkonen, Bradley Pearce, Michael Rudd, Annette Werner, Bei Xiao

**Affiliations:** Durham University, UK; Columbia University Irving Medical Center, United States; New York State Psychiatric Institute, United States; Teachers College Columbia University, United States; Institute of Biosciences, Newcastle University, UK; Durham University, UK; University of Helsinki, Finland; Institute of Neuroscience, Newcastle University, UK; University of Nevada, United States; Max Planck Institute for Biological Cybernetics, Germany; American University, United States

**Keywords:** #thedress, color perception, spatial context, chromatic illumination, color constancy, material–light interactions

## Abstract

The colors that people see depend not only on the surface properties of objects but also on how these properties interact with light as well as on how light reflected from objects interacts with an individual’s visual system. Because individual visual systems vary, the same visual stimulus may elicit different perceptions from different individuals. #thedress phenomenon drove home this point: different individuals viewed the same image and reported it to be widely different colors: blue and black versus white and gold. This phenomenon inspired a collection of demonstrations presented at the Vision Sciences Society 2015 Meeting which showed how spatial and temporal manipulations of light spectra affect people’s perceptions of material colors and illustrated the variability in individual color perception. The demonstrations also explored the effects of temporal alterations in metameric lights, including Maxwell’s Spot, an entoptic phenomenon. Crucially, the demonstrations established that #thedress phenomenon occurs not only for images of the dress but also for the real dress under real light sources of different spectral composition and spatial configurations.

In 2015, an image of a dress (#thedress) went viral as people divided roughly into two populations, depending on how they named its colors: blue/black or white/gold. A plausible explanation proposed for this split is that individuals differ in the way their visual systems assign probabilities to different illuminations when estimating surface color (see e.g., Brainard & Hurlbert, 2015; Gegenfurtner et al., 2015; Lafer-Sousa & Conway, 2017, Wallisch, 2017; Witzel et al., 2017). Color constancy is the perceptual phenomenon by which perceived object colors remain approximately stable under changes in illumination (c.f. Hurlbert, 2007; Monge, 1789; Shepard, 2001; von Helmholtz, 1867). It is a prime illustration of how the human visual system resolves uncertainties in the incoming sensory signals to construct robust representations of object properties (Brainard & Radonjić, 2014; Foster, 2011; Hurlbert, 1998; Olkkonen & Ekroll, 2016; Smithson, 2005; Xiao, 2020). The incoming information from #thedress image is ambiguous: Cues to the physical characteristics of the illumination are sparse yet conflicting, and the dress is an unfamiliar object with an unknown surface reflectance, conferring similar likelihoods to distinct combinations of surface and illumination properties consistent with the incoming image. Furthermore, the image chromaticities are distributed in a particular way, aligning roughly with the daylight locus, amplifying the uncertainty that chromaticity variations in the image arise from material variations in the object (e.g., Gegenfurtner et al., 2015). Quantitative empirical studies demonstrate that reported colors do not fall exclusively into binary categories when people are allowed free naming of the dress, but they do differ significantly between individuals (Aston & Hurlbert, 2017; Lafer-Sousa & Conway, 2017; Werner, 2015; Werner & Schmidt, 2016), and individual differences in reported dress colors do indeed vary with individual differences in perceived illumination colors (Aston & Hurlbert, 2017; Toscani et al., 2017; Uchikawa et al., 2017; Wallisch, 2017; Witzel et al., 2017). These results support the color constancy explanation, that is, that differences in disambiguating surface reflectance versus illumination spectrum underlie differences in #thedress color perception. People who see the dress as white/gold tend to perceive the illumination in the photo as bluer and darker, whereas people who see the dress as blue/black see the illumination as yellower and brighter. Other studies demonstrate that providing additional cues to the illumination spectrum may drive individual perceptions toward a particular naming category (Witzel et al., 2017), as do image manipulations such as illusory brightness changes (Hugrass et al., 2017), spatial filtering (Dixon & Shapiro, 2017), or spatial occlusion (Daoudi et al., 2017).

Although all such reported studies have been performed with two-dimensional images only, several unpublished public demonstrations have shown that the ambiguity of #thedress may be achieved in a real scene. When the real dress (*blue colorway*^1^) is illuminated simultaneously by two light sources, one blueish and one yellowish, its appearance differs from that under a single white light, and people disagree on its color. In one such demonstration^2^, the real dress was simultaneously lit by a diffuse blue light (chromaticity CIE *x*,*y* = 0.253, 0.274) and a more directed yellow light (mimicking candle light; chromaticity CIE *x*,*y* = 0.459, 0.407), both illuminations generated by tuneable multichannel LED lamps (www.hi-led.eu and www.ledmotive.com). Under these conditions, the proportions of blue/black versus white/gold perceivers were found to be similar as for the original photo (#thedress). But when free naming was allowed, the variety of dress color names increased (e.g., for a population of 847 individuals, the split was 46% blue/black, 12% white/gold, and 42% other under the ambiguous two-source lighting, vs. 86% blue/black, 2% white/gold, and 12% other, for a single-source white light^3^). Here, we report a series of demonstrations using real materials in three dimensions, including the real dress, presented at VSS 2015, which probe the principles underlying the individual variability of color perception under changing illumination spectra. These demonstrations illustrate that targeted manipulation of the spectral content, spatial distribution, and temporal dynamics of the illumination affects people’s perception not only of object colors but also of their material properties more generally. The demonstrations also confirm, crucially, that #thedress phenomenon occurs not only for the photographic image but also for the real dress under real light sources of different light colors, and that manipulations of physical features such as the background, other contextual objects, and illumination spot size may drive changes in individual dress color percepts.

## The Setup

Demos were presented in a single, large room, at four different stations along three walls. The demos ran for 5 hours on the evening of May 18, 2015. Approximately 1,500 people in total attended, each individual typically spending 5 to 10 min at each station.

## Demo 1: The Real Dress Changes Color: Effects of Chromatic Context and Multiple Illuminations

To resolve image ambiguities, the human visual system must employ constraints, such as assumptions about the probability of particular environmental conditions, based on prior experience (Knill & Richards, 1996; Yuille & Kersten, 2006) and biological plausibility (Shepard, 2001; Denisova, Feldman, et al., 2016). In computational models of color constancy, one such constraint is the *single-source assumption* (Brainard et al., 2006; Hurlbert, 1998): disentangling surface reflectance from the illumination spectrum in the reflected light signal becomes more feasible if the illumination spectrum is assumed to be spatially uniform.

In real scenes, such as the one that gave rise to #thedress, there is likely to be more than one light source, of different spectra, and in different locations (e.g., shadow in the foreground, direct light from behind). Thus, the single-source assumption is violated. To achieve color constancy in such scenes, the visual system must accurately register the light field (the distribution of the illumination across the scene), using cues or priors for image segmentation (Werner, 2014), as well as for interpreting the spatial layout of the scene (Bloj et al., 1999). #thedress phenomenon suggests that in the absence of *hard* cues, different individuals use different assumptions about the scene and its illumination to resolve the ambiguity inherent in the image.

**Figure 1.**
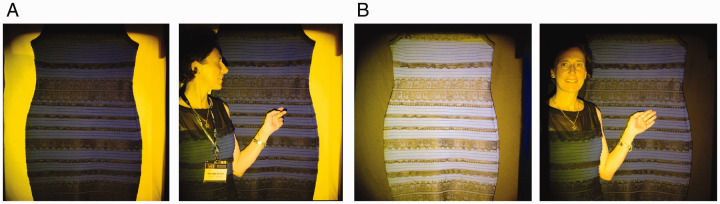
Contextual effects. The real dress (which under white light appears blue [body]/black [lace]) is shown on a poster board covered with (A) yellow fabric, or (B) black fabric, in both cases illuminated by a mixture of blue and yellow light. The presence of a person in the scene affects the dress color in both conditions. Model: Annette Werner.*Note.* Please refer to the online version of the article to view the figure in colour.

Here, we demonstrated that changes in the light field and spatio-chromatic context of the real dress altered viewers’ perceptions of its colors, causing these to vary from blue/black to lavender/brown to white/gold, the same alternatives reported for #thedress image. In the first of these manipulations, we displayed a hanging version of the real dress^1^ against either a nearly black or yellow cloth background and illuminated the scene with a mixture of two broadband chromatic lights (yellow and blue) from two slide projectors. In this set up, the dress appeared to most observers as blue/black (Figure 1A) when presented against the yellow background, whereas it was white/gold for most observers when viewed against the black background (Figure 1B). Thus, the chromatic context of the real dress strongly influenced its reported color. When quantifying this effect later under controlled lab conditions, it was found that this change affected the original blue/black viewers more strongly (i.e., inducing a larger color shift) than it did the original white/gold viewers (Werner, 2015; Werner & Schmidt, 2016). In other words, the contextual effect differed between the different perceptual groups and increased the ambiguity. Therefore, these contextual effects go beyond the previously reported importance of context for the emergence of the ambiguity (e.g., Hesslinger & Carbon, 2016; Jonauskaite et al., 2020; Witzel et al., 2017; Witzel, Poggemann et al., 2017). Other related experiments in the Werner laboratory demonstrated that the differential effect of the background color is not explained by chromatic induction alone since neither the viewing behavior of the subjects (time spent viewing the dress or the background) nor the strength of induction in general (as measured on a display in a center-surround paradigm) differed accordingly between the observers (Werner & Schmidt, 2016; Weigold, 2017).

**Figure 2.**
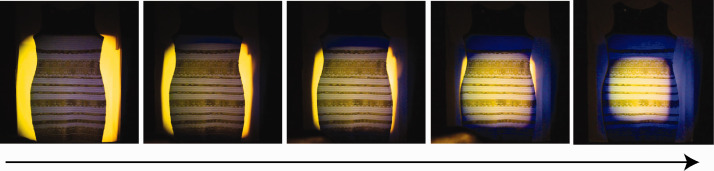
Spotlight size manipulation affects perceived dress color. The real dress is displayed against a yellow cloth background. In the sequence of images from left to the right, the yellow illumination is increasingly restricted to the center of the dress, with the blue illumination falling on its outer portions. In the final, right-most image, the center of dress is illuminated by the mixture of yellow and blue light, the edges of dress illuminated by blue light only.*Note.* Please refer to the online version of the article to view the figure in colour.

In the second manipulation, we changed the color of the illumination specifically in one part of the scene: When the illumination on the background and outer dress portion was changed from a mixture of yellow and blue to blue only, people were pushed into seeing the remaining, fully lit part of the dress as white/gold (Figure 2). Thus, this demo showed that the dress color is influenced both by the chromaticity of the illumination and also—very strongly—by its spatial distribution, the light field. Similar effects have been reported for manipulations of the original photograph (Witzel et al., 2017).

We also demonstrated other contextual effects that had not been previously noted: Changes in the perceived dress color were evoked by introducing a real person or a white reference paper into the scene, under the same spotlight illuminating the dress. For example, Figure 1 illustrates the effect of having a real person in the scene—in this case, wearing the real dress. Against either background, the presence of a real person pointing to the dress nudges its perceived color toward blue/black. We also noted novel slow color recalibration effects, evolving on the order of several seconds, following the removal of the background anchor or the reference object from the illumination frame. To our knowledge, slow temporal adaptation to changes in lightness and color anchoring have not been previously noted, let alone systematically studied.

In summary, these effects demonstrate a strong role for the chromatic context and light field in determining the perceived color of the real dress. The contextual effects may be summarized in the framework of the color constancy explanation for #thedress phenomenon. To interpret the dress color, the visual system must simultaneously interpret the illumination, yet there are conflicting cues. The background color provides a strong cue—particularly under the gray-world assumption (Hurlbert, 1998)—and given the uncertainty over the number and type of light sources, the visual system puts particular weight on the information it conveys. Hence, changing the background color may alter the unconsciously estimated illumination color and thereby the dress color: the yellow background signals the presence of a yellow illumination, compensation for which yields a blue/black dress; also, at the same time, this background becomes the brightest surface in the scene, and thus, consistent with the *anchoring* rules described for lightness perception (Gilchrist, 2006; Gilchrist et al., 1999; Gilchrist & Soranzo, 2019; Rudd, 2017, 2020; Rudd & Zemach, 2005; Werner, 2015; Werner & Schmidt, 2016), becomes a strong reference cue for the illumination. Conversely, the nearly black background signals a neutral, low-intensity illumination, compensation for which yields a brighter, white/gold dress (later measurements of the same real dress under comparable illumination in the laboratory revealed that its chromaticities were in fact close to achromatic). The effects of adding a real person to the scene are most likely explained by the presence of human skin—a familiar object with known surface reflectance—providing an additional reference surface from which the illumination chromaticity may be inferred (Crichton et al., 2012). The observed ambiguity of #thedress phenomenon can be explained by individual variations in the degree to which the background is used as a reference by the different observers (Werner & Schmidt, 2016).

## Demo 2: The Real Dress: Effects of Chromatic Illuminations

Another assumption that enables solutions to the computational problem underlying color constancy is that the light source spectrum is broadband, with no gaps in power across the visible range of wavelengths (Brill, 1978; Hurlbert, 1998). Natural daylight and incandescent light satisfy this assumption; narrowband, highly chromatic illuminations do not. The latter generate ambiguous reflected light signals from surfaces, containing sparse information about surface reflectance. This demo illustrated the ambiguity arising from such atypical illuminations, the aim being not to reproduce the specific illumination conditions of #thedress, but to illustrate the challenge posed to color constancy by extreme violations of the single and broadband source assumptions.

**Figure 3.**
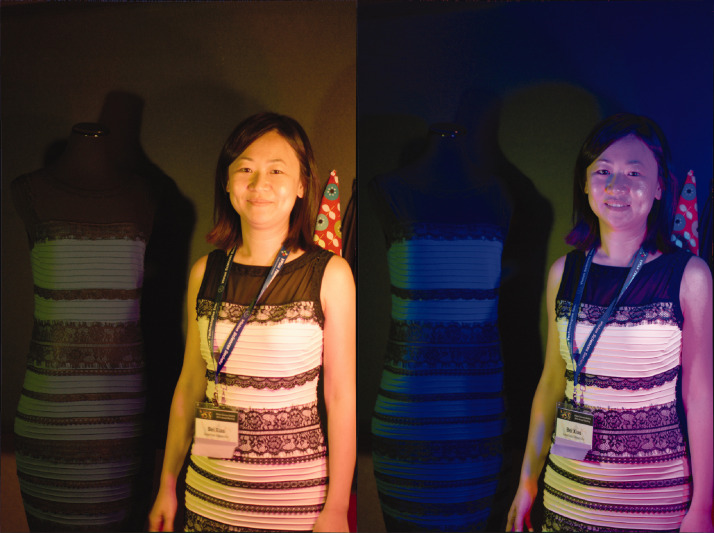
A comparison of perceived color of the blue/black and the white/black dress. The blue/black and white/black dresses are illuminated by two different illuminants in the left and right panels. The color appearance of the blue/black dress varies under changing illumination (compare the dress shown on the left across both panels). In contrast, the white dress seems to vary less in color appearance under the same illumination changes (relative to the dress on the left in both panels). Most observers reported the blue/black dress as changing more in color appearance than the white/black dress. Model: Bei Xiao.*Note.* Please refer to the online version of the article to view the figure in colour.

The real dress^1^ (*blue colorway*) was illuminated simultaneously by three tuneable four-primary LED sources (www.milight.com), spatially separated. The output of each source was varied smoothly and randomly over time, asynchronously, to produce spatially and spectrally mixed illuminations spanning multiple directions in color space. Thus, the illumination was extremely unnatural: multiple light sources, each with a highly chromatic spectrum, changing randomly over time. As the illuminations changed, so did the apparent color of the dress, demonstrating the failure of color constancy mechanisms under these conditions. Yet when we added to the scene the white/black version of the real dress (the alternative *Ivory colorway* supplied by *Roman Originals*^1^), kindly modeled by a fellow demonstrator, we were also able to illustrate how color constancy under these atypical illumination conditions is worse for chromatic than achromatic surfaces (Figure 3). Interestingly, for less extreme illumination conditions, constancy as measured by consistency of color naming under changing illumination is generally found to be similar for chromatic and achromatic surfaces (Olkkonen et al., 2009, 2010; Troost & de Weert, 1991).

**Figure 4.**
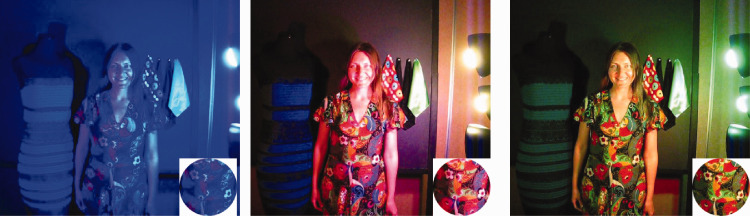
Illustration of relatively local color constancy effects under changing illuminants in the case of a multicolor dress. Note that the white flower (inset) remains a relatively constant color in contrast to other parts of the dress containing flowers of varying colors. Model: Kristina Denisova.*Note.* Please refer to the online version of the article to view the figure in colour.

In this demo, viewers consistently stated that the dress body of the *ivory* dress was white. In fact, the color of the *ivory* dress appeared so stable in this demo that had this been the dress chosen that day in the shop, the Internet phenomenon might never have happened. The presence of the white dress also helps to stabilize the color of the blue/black dress under extreme illumination changes: its color seems to change less when the two dresses are shown side-by-side under the same changing illumination. These results point to the explanation that the *ivory* dress, as the brightest surface in the scene, serves as an anchor, or a reference surface from which the illumination chromaticity may be estimated. This explanation fits the third possible assumption employed by the human visual system to resolve the computational problem underlying color constancy: the *brightest-is-white* assumption (Brenner & Nascimento, 2012; Hurlbert, 1998; Rudd, 2013, 2017, 2020; Rudd & Zemach, 2005). Note that a more local, but similar, white constancy effect can be observed in the case of a dress made from multicolor fabric (Figure 4).

The stability of the *ivory* dress under extreme changes in illumination supports the hypothesis that the particular surface properties of the real dress are partly responsible for #thedress phenomenon. These properties cause the image chromaticities to vary from bluish to brownish-yellow, along the daylight locus, lending plausibility to the assumption of a bluish or yellowish daylight illumination (Gegenfurtner et al., 2015; Lafer-Sousa et al., 2015; Winkler et al., 2015). Indeed, recent work demonstrates that the particular image color distribution of #thedress, including both luminance and chromaticity components, is sufficient to elicit individual differences in perception when transferred to images with other content (Witzel & Toscani, 2020). #thedress phenomenon, and these demos, show that color constancy is not all-or-none, but depends on the particular surface reflectance and illumination spectrum combinations (see also Aston & Hurlbert, 2017; Hurlbert, 2019).

## Demo 3: Material–Light Interactions Beyond #thedress

We demonstrated how our visual system employs various cues in our environment, such as material properties, scene contexts, and fabric pigments, when inferring the surface color of objects. In this demonstration, a set of fabric swatches were placed next to the real dress^1^ (*blue colorway*) on a mannequin. Again the three tuneable four-primary LED sources, spatially separated, provided atypical illumination, with the illumination spectrum from each source changing randomly and independently in time.

**Figure 5.**
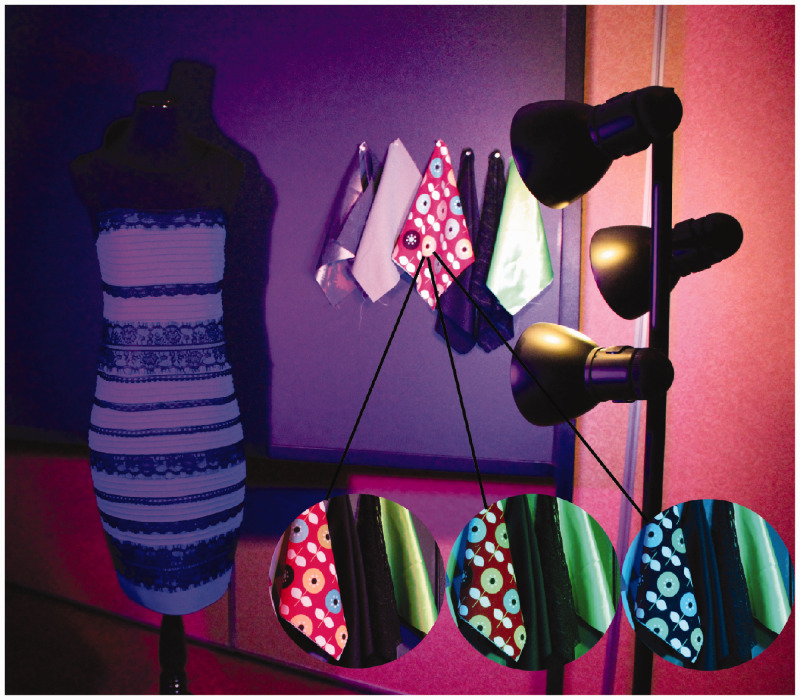
The dress and other fabrics were displayed under smoothly changing chromatic illumination. The blue/black dress appears in different colors to different people under different chromatic illuminations. Perceived color also changes dramatically for some fabrics, such as the corduroy floral and the shiny metallic swatches. The material properties of fabrics affect the colors we see under varying illumination.*Note.* Please refer to the online version of the article to view the figure in colour.

As the illumination on the fabrics changed over time, the appearance of certain fabrics changed even more dramatically than the blue/black dress (Figure 5). The changing appearance of these fabrics illustrated that the instability in surface colors under changing illuminations is not specific to the dress, but occurs for a variety of fabrics. The corduroy fabric with colored flowers changed in color appearance from bright red to black. If the viewer focused only on this particular fabric, he or she would have difficulty in recovering its color appearance when viewed under broadband light (which the viewer would consider the *real* color of the fabric). The shiny silk also changed its color appearance dramatically as the color of the light source changed. In addition, its material properties seemed also to change from plastic to metallic.

This demonstration shows that there is a strong interaction between illumination and material properties in influencing the color appearance—and color constancy—of fabrics. Note that while the color of fabrics has been studied from the physics perspective (e.g., Allen & Goldfinger, 1972), this subject is a completely understudied area from the perception perspective. The physics of how light interacts with fabrics is complex, involving processes of absorption, subsurface scattering, transmission, and reflectance; these processes all depend on the spectrum of the illumination (Zhao et al., 2011). The interaction of material with light depends on the material properties of the fabric, such as the *looseness* of the knit (i.e., how densely the fibers are woven together) and the specific constitution of the fibers (e.g., natural wool fibers or polyester fibers; Zhao et al., 2012). However, how the material properties of fabrics are perceived might affect how their color is perceived, and vice versa (Xiao et al., 2016). A recent study examined the relationship between material perception and perceived mode of color appearance (Kuriki, 2015). Using either matte gray objects or objects with fabric textures, Kuriki found that luminosity (mode) could be strongly affected by the material percept. Another recent study, on the other hand, showed that both surface glossiness and surface diffuse reflectance influenced whether the surface would be categorized as *gold* or *silver*, which indicates that humans do not always discount surface gloss to identify colors but can utilize this information to categorize surface colors (Okazawa et al., 2011). However, our demo shows that more work is needed to investigate the relationship between material perception and color perception with complex materials such as fabrics.

In this demo, we found perceptual ambiguity not only in color perception but also in material perception under varying lighting conditions. The color changes co-occurred with changing material appearance. #thedress phenomenon opens new doors for studying material perception under real lighting and for understanding individual differences. It is possible that material categorization affects material perception. For example, the particular combination of surface glossiness and the color appearance of the lace probably contributed to its color categorization and metallic appearance.

## Demo 4: Magical Metamers Light Show

A spectrally nonselective surface—that is, one that reflects equally all wavelengths in the visible spectrum and appears *white*—will perfectly reflect the incident illumination (neglecting geometrical and scene configuration effects). Thus, the illumination chromaticity may be estimated directly from the chromaticity of white surfaces. This notion underpins *white-balancing* methods used to calibrate digital cameras (e.g., Akkaynak et al., 2014) and combined with the *brightest-is-white* assumption (Brenner & Nascimento, 2012; Hurlbert, 1998; Rudd, 2013, 2017, 2020; Rudd & Zemach, 2005) provides a method for estimating the illumination chromaticity when there is no *a priori* identification of a perfectly spectrally nonselective surface. The varieties of colors seen in #thedress phenomenon may arise from different people *white-balancing* to different parts of the image, and thereby estimating and correcting for different illumination chromaticities.

In the Magical Metamers Light Show, we showed that white-balancing is not a failsafe mechanism for color constancy, using contemporary lighting technology that challenges the human visual system’s assumptions about typically occurring illuminations.

**Figure 6.**
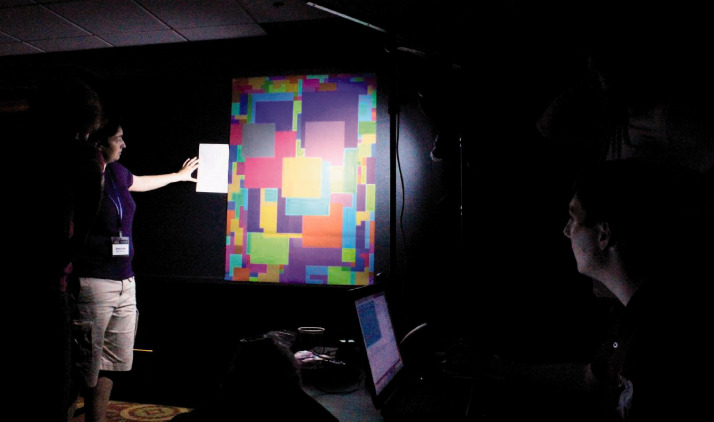
Illustration of the magical metamers setup. Experimenters: Stacey Aston, Brad Pearce, and Anya Hurlbert.*Note.* Please refer to the online version of the article to view the figure in colour.

A white tile and a large *Mondrian* print were pinned to a black display board and illuminated by a single tuneable multichannel LED luminaire (www.hi-led.eu and www.led-motive.com; Figure 6). The luminaire is able to produce, under real-time computer control, illumination of almost any desired spectra—from daylight, to candlelight, to fluorescent light.

**Figure 7.**
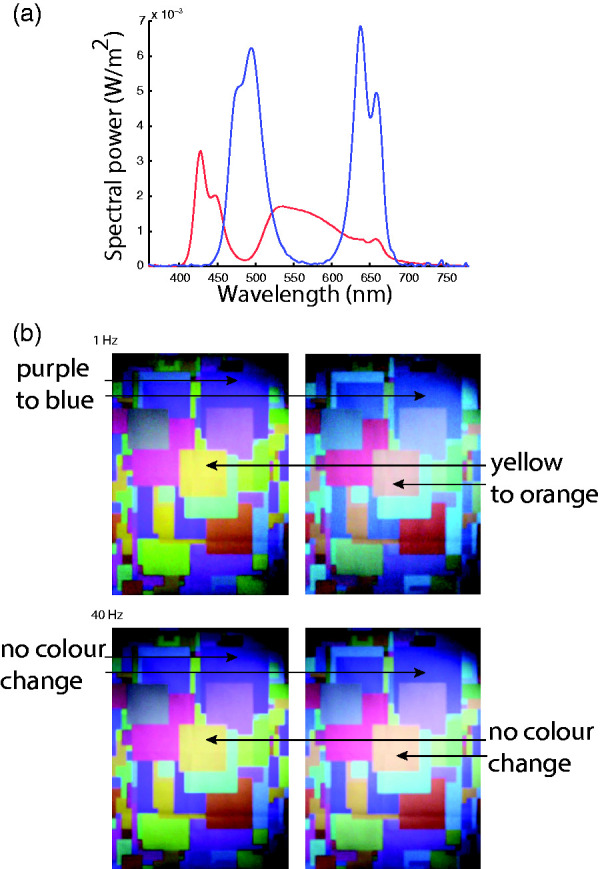
White-Balancing and the role of exchange rate of illumination. At relatively slow alternations (1 Hz) (B, upper) between the two illuminations whose spectra are shown in Panel A, observers perceive a change in tiles, in particular, the yellow center tile. At faster exchange rates (B, lower), this percept no longer occurs; instead of a specific tile switch, a flicker over the entire panel can be seen.*Note.* Please refer to the online version of the article to view the figure in colour.

The Mondrian and tile were illuminated by two distinct light spectra, produced in temporal alternation at about 1 Hz by the single luminaire. The two light spectra were metamers, calculated to elicit the same triplet of responses from the three cone types (L, M, and S) according to the CIE (2006) two-degree color matching functions (Figure 7). So the white tile appeared not to change color; it remained white throughout. The surfaces in the Mondrian, however, radically changed color under the two metameric illuminations (Supplemental Video 1). Orange changed to yellow; blue changed to lilac (Figure 7, top panel). Because the Mondrian surfaces do not reflect equally across the spectrum, as the white tile does, they reflect the two different spectra of metameric lights differently. The human visual system interprets these changes in the reflected light as a change in the actual surfaces of the Mondrian, because such changes in reflected light are rare in nature. *White-balancing* fails because the two lights appear the same when reflected from the white tile. The lights stay the same color, but the surface colors change: a rare failure of color constancy.

When the exchange rate of the two metameric lights is increased to 40 Hz, the surface colors no longer appear to change, but instead there is a visible light flicker (Figure 7 bottom panel; Supplemental Video 1). The human visual system seems to switch to a mode of perceiving an illumination change instead of a surface color change, merely through an increase in the rate of illumination change. This phenomenon, of the temporal frequency dependence of surface versus illumination change perception, differs from the chromatic fusion observed in the traditional heterochromatic flicker photometry (HFP) paradigm (Lennie et al., 1993) in key ways: (a) above the critical chromatic fusion frequency, the HFP stimulus is perceived as unitary, whereas here it is binary, dividing into percepts of a physical surface *beneath* a distinct illumination; (b) unlike the traditional HFP stimulus, here the stimulus does not merge into a single hue with flickering intensity but retains its complexity of multiple, differently colored surfaces; and (c) the illumination over the whole scene appears to remain spatially uniform, contrary to what would be predicted if each surface patch behaved like a distinct HFP stimulus. The phenomenon is currently under further investigation in the Hurlbert laboratory.

This demonstration shows that estimating the illumination chromaticity from a white surface does not reliably achieve color constancy; surface colors may radically differ even for the same estimated illumination chromaticity. Again, as does #thedress, this phenomenon illustrates that color constancy depends on multiple mechanisms, which may yield different results depending on the particular materials and illumination spectra involved. Different individuals may also weight the contributing mechanisms differently, depending on their previous experiences and prior expectations, leading to #thedress-type debates.

## Demo 4.1: See Your Very Own Maxwell’s Spot

The temporal alternation between two metameric lights (Figure 7A) provided an added bonus: attendees were able to see their own *Maxwell’s spot* (Maxwell, 1856/1857; Flom & Weymouth, 1961). Viewers were asked to fixate on the white tile. As the light changed between the two metamers, the tile stayed the same in color, as it reflected the two spectra faithfully. Yet a large spot of color was visible in the center of each viewer’s vision, alternating between pink and green. This entoptic phenomenon, Maxwell’s spot, is caused by the macular pigment differentially absorbing more short-wavelength (blue) light than the other retinal layers surrounding the fovea, resulting in the macular cones receiving different input in comparison with the surrounding cones, when all are stimulated by the same uniform field of light. Thus, the two lights will differ in the degree of metamerism they achieve in the unfiltered peripheral versus macular-pigment-filtered foveal cones, and therefore, in the degree of change they elicit in the cone response. The change in color of the central spot is therefore formed by the change in contrast between center and peripheral stimulation.

## Conclusions and Implications

The demos presented at the 2015 Vision Sciences Society Dress Pavilion illustrate using real materials and lights that how #thedress is perceived depends on the illumination impinging on the dress as well as its spatio-chromatic context. This provides support for the hypothesis that the different perceptions of the dress depend on different interpretations of the illumination, within a color constancy framework in which the visual system adopts particular assumptions to resolve ambiguities due to uncertain image information (e.g., Chetverikov & Ivanchei, 2016; Lafer-Sousa et al., 2015; Toscani et al., 2017; Wallisch, 2017; Werner, 2015; Werner & Schmidt, 2016; Witzel et al., 2017). Our demos extend this observation to other fabrics as well, paving the way for the study of the perception of real materials under real illuminant changes.

We show with the original blue/black dress that changing the background of the dress from black to yellow, or the light illuminating the dress from bluish to yellowish, has a dramatic effect on the appearance of the dress. This effect is complementary to the original #thedress phenomenon in which different individuals experience different percepts when observing the *same* image of the dress, owing to the ambiguity of the image with respect to the underlying scene and its illumination. In our Demo 1, the same observer experienced different percepts when observing the real dress in different visual scenes, containing different reference cues. Interestingly, having an unambiguous reference, that is, an unambiguous background or familiar object, seemed to stabilize the perception of the dress color—the *overexposed* dress under the bright spotlight looks more blue/black when skin is visible in the scene. This effect provides tentative evidence for the effect of anchoring to a familiar color (Brenner & Nascimento, 2012). Our other demos showed conflicting evidence for anchoring: on one hand, the *ivory* dress seemed to remain much more perceptually stable under extreme changes in illumination compared to the blue/black dress, while on the other hand, having it nearby did not make the blue/black dress substantially more perceptually stable. The substantial difference in the illumination conditions in the two cases (Demo 1 vs. Demo 2) may partially account for this conflict. The Magical Metamers demonstration further showed the limits of the anchoring account; although there were white surfaces in the scene, they did not counteract the effect of changing illumination on the color appearance of chromatic surfaces, which seemed to change color at particular frequencies of illumination change. Understanding how the visual (and more broadly, perceptual) system balances and combines weak, conflicting, or incomplete types of information to maintain stable representations of the environment is critical for understanding not only color but also other perceptual attributes. Understanding the interactions of bottom-up sensory processing with prior knowledge, and whether and how such priors are represented neurally, is also critical to understanding neurodevelopmental disorders, such as autism spectrum disorder, in which these interactions develop atypically (Denisova, 2019; Denisova, Zhao, et al., 2016).

Taken together, our demonstrations offer both support and rebuttal to theories and models of color constancy developed to explain the perception of color in simpler scenes. These demos, while not performed in controlled laboratory conditions, highlight the need to study color constancy for complex surfaces and illuminants, while broadening focus from diffuse reflectance to other properties of surface materials. Although there is little research on color constancy with real (or realistically rendered) polychromatic surfaces or surface textures, other studies indicate that naturalistic textures and shapes influence color perception of objects (Olkkonen et al., 2008; Vurro et al., 2013). The interactions between material and color perception illustrated by our demonstrations provide further motivation for probing the rich interactions between intrinsic and extrinsic factors that contribute to the diversity of individual perceptual inference and experience.

## Authors' note

Stacey Aston is currently affiliated with the Department of Psychology, Durham University, Durham, DH1 3LE, UK.

Correspondence may be addressed to the following authors:

Stacey Aston, Department of Psychology, Durham University, Durham, DH1 3LE, UK.

Email: stacey.j.aston@durham.ac.uk

Kristina Denisova, Sackler Institute for Developmental Psychobiology and Department of Psychiatry, Columbia University College of Physicians and Surgeons, New York, NY 10032, USA; Division of Developmental Neuroscience, New York State Psychiatric Institute, New York, NY 10032, USA; Biobehavioral Sciences Department, Teachers College, Columbia University, New York, NY 10027, USA.

Email: kd2401@tc.columbia.edu

Anya Hurlbert, Neuroscience, Institute of Biosciences, Newcastle University, Newcastle upon Tyne, NE2 4HH, UK.

Email: anya.hurlbert@newcastle.ac.uk

Maria Olkkonen, Department of Psychology, Durham University, Durham, DH1 3LE, UK; Department of Psychology and Logopedics, Faculty of Medicine, University of Helsinki, 00014 University of Helsinki, Finland.

Email: maria.olkkonen@helsinki.fi

Michael Rudd, Department of Psychology and Center for Integrative Neuroscience, University of Nevada, Reno, NV 89557, USA.

Email: mrudd@unr.edu

Annette Werner, Department for Physiology of Cognitive Processes, Max-Planck-Institute for Biological Cybernetics, D-72076, Tübingen, Germany.

Email: annette.werner@mpg.tuebingen.de

Bei Xiao, Department of Computer Science, American University, Washington, DC 20016, USA.

Email: bxiao@american.edu

## Notes

1 The dress was made by Roman Originals©; PLC Registered office: Unit 1, Vantage Point, 5 Wingfoot Close, Birmingham, B24 9JH.

2 This demonstration was run as a mass experiment in the “Some Like Dark” show that ran during the Wellcome Collection’s “On Light” weekend, May 1–4, 2015 (produced by Hurlbert, Pearce, and Aston, Newcastle University). It was also shown in BBC4’s programme, “Colour: The Spectrum of Science,” Episode 3, Beyond the Rainbow. https://www.bbc.co.uk/programmes/b06pm7t8, November 2015.

3 Unpublished data, collected during the Wellcome Collection’s “On Light” weekend, May 1–4, 2015 (Hurlbert, Pearce, and Aston, Newcastle University). See also Hurlbert et al. (2015).
